# DISPARITIES IN HEALTH OUTCOMES AMONG MIDDLE-AGED AND OLDER SEXUAL MINORITY CANCER SURVIVORS

**DOI:** 10.1093/geroni/igae098.2485

**Published:** 2024-12-31

**Authors:** Maria Brown, Jane McElroy

**Affiliations:** Syracuse University, Syracuse, New York, United States; University of Missouri, Columbia, Missouri, United States

## Abstract

This cross-sectional, exploratory study analyzed 2021 Behavioral Risk Factor Surveillance System public-use data for a propensity-score matched sample (N=25,545), from states that collected sexual identity and cancer survivorship information, to explore health outcomes among sexual minority (SM) and non-SM cancer survivors. In bivariate analysis, SM survivors had more chronic conditions than non-SM survivors (males 45-64 years: 1.67 vs 1.03; females 45-64 years: 1.46 vs 1.16) and were more likely to report multiple chronic conditions (males 45-64 years: 44.5% vs 29.0%), depressive disorders (males 45-64 years: 42.9 vs 23.4%; males 65+ years: 20.8% vs 11.2%; females 45-64 years: 52.9% vs 36.5%; females 65+ years: 42.7% vs 41.3%), and difficulty concentrating, remembering, or making decisions because of a physical, mental, or emotional condition (females 45-64 years: 30.1% vs 18.9%; males 65+ years: 15.9% vs 8.5%). In logistic regression models, after controlling for demographics, health, and health behaviors, SM males and females in both age groups had similar odds of being a cancer survivor as their non-SM counterparts, and greater odds of poor health, multiple chronic conditions, depressive disorder, ADL limitations, and difficulty concentrating, remembering, or making decisions. Cancer status was not always associated with these outcomes. Further research should explore treatment experiences and factors contributing to disparities within this population and among SM cancer survivors and assess whether health outcomes of SM cancer survivors are related to stage of disease at diagnosis, time since treatment, or decisions made during treatment.

